# Under-utilisation of noncommunicable disease screening and healthy lifestyle promotion centres: A cross-sectional study from Sri Lanka

**DOI:** 10.1371/journal.pone.0301510

**Published:** 2024-04-04

**Authors:** Thilini Herath, Manuja Perera, Anuradhani Kasturiratne

**Affiliations:** 1 Faculty of Health-Care Sciences, Department of Primary Health Care, Eastern University, Batticaloa, Sri Lanka; 2 Faculty of Medicine, Department of Public Health, University of Kelaniya, Ragama, Sri Lanka; Kasturba Medical College Mangalore, Manipal Academy of Higher Education, INDIA

## Abstract

**Background:**

Healthy Lifestyle Centres (HLCs) are state-owned, free-of-charge facilities that screen for major noncommunicable disease risks and promote healthy lifestyles among adults older than 35 years in Sri Lanka. The key challenge to their effectiveness is their underutilisation. This study aimed to describe the underutilisation and determine the factors associated, as a precedent of a bigger project that designed and implemented an intervention for its improvement.

**Methods:**

Data derived from a community-based cross-sectional study conducted among 1727 adults (aged 35 to 65 years) recruited using a multi-stage cluster sampling method from two districts (Gampaha and Kalutara) in Sri Lanka. A prior qualitative study was used to identify potential factors to develop the questionnaire which is published separately. Data were obtained using an interviewer-administered questionnaire and analysed using inferential statistics.

**Results:**

Forty-two percent (n = 726, 95% CI: 39.7–44.4) had a satisfactory level of awareness on HLCs even though utilisation was only 11.3% (n = 195, 95% CI: 9.80–12.8). Utilisation was significantly associated with 14 factors. The five factors with the highest Odds Ratios (OR) were perceiving screening as useful (OR = 10.2, 95% CI: 4.04–23.4), perceiving as susceptible to NCDs (OR = 6.78, 95% CI: 2.79–16.42) and the presence of peer support for screening and a healthy lifestyle (OR = 3.12, 95% CI: 1.54–6.34), belonging to the second (OR = 3.69, 95% CI: 1.53–8.89) and third lowest (OR = 2.84, 95% CI: 1.02–7.94) household income categories and a higher level of knowledge on HLCs (OR = 1.31, 95% CI: 1.24–1.38). When considering non-utilisation, being a male (OR = 0.18, 95% CI: 0.05–0.52), belonging to an extended family (OR = 0.43, 95% CI: 0.21–0.88), residing within 1–2 km (OR = 0.29, 95% CI: 0.14–0.63) or more than 3 km of the HLC (OR = 0.14, 95% CI: 0.04–0.53), having a higher self-assessed health score (OR = 0.97, 95% CI: 0.95–0.99) and low perceived accessibility to HLCs (OR = 0.12, 95% CI: 0.04–0.36) were significantly associated.

**Conclusion:**

In conclusion, underutilisation of HLCs is a result of multiple factors operating at different levels. Therefore, interventions aiming to improve HLC utilisation should be complex and multifaceted designs based on these factors rather than merely improving knowledge.

## Introduction

One of the vital, cost-effective strategies in preventing NCDs is screening and proper management of people at risk. The main aim of those services is to prevent the progression of common NCDs and reduce the associated burden of disease and death [1]. World Health Organization’s (WHO) essential intervention package recommends integrating with primary care to ensure the participation of poor and most vulnerable individuals and communities [1]. This strategy aims to reduce inequality by improving access and affordability for the needy and high-risk people. However, the success of a screening program is also dependent on public participation and the reach of the service to the target population [2]. According to previous worldwide studies, uptake of state screening services by asymptomatic individuals is significantly low [2–5].

Healthy Lifestyle Centres (HLCs) in Sri Lanka, which is the first such initiative in Southeast Asia, is a response to promote early detection and management of Noncommunicable Diseases (NCDs) and risk factors at the Primary Health Care (PHC) level [6]. HLCs aim to cater for the poorest societal segments aiming to prevent Cardio Vascular Diseases (CVDs) [7]. The primary target population for HLCs is adults aged over 35 years who are not diagnosed with any form of NCD. Ministry of Health expects HLCs to function at least once per week from 8.00 am to 12.00 noon and the services are offered absolutely free of charge. Self-referral is promoted via health education through posters, banners, leaflets, printed invitations, health talks, and referrals by field health staff and medical officers. Body mass index (BMI), waist circumference, blood pressure, capillary fasting blood sugar, and total cholesterol are among the measurements taken at the HLCs, and clients will also receive lifestyle modification advice and a date for the follow-up visit based on the 10-year CVD risk [8].

Similar to the global context, HLCs in Sri Lanka report a low uptake by its potential clients. Even though HLCs are financially and physically available for all community segments, according to the data of the pre-pandemic era (2018 and 2019), the reported utilisation rate of HLCs was only 10.0% and 6.9% respectively across the country. Further, there was substantially low male participation (male-to-female ratio of 1:2.2 (2018) and 1:2.6 (2019)) [9,10]. The annual utilisation has been reduced further to 3.7% in 2020 and 2.9% in 2021 with the COVID-19 pandemic [9]. During the first quarter of 2022, the utilisation has further reduced to 1.5% [11]. Therefore, the overwhelming challenge faced by the HLC service providers is underutilisation [12–14].

According to the national multisectoral action plan for the prevention and control of NCDs [15], it is targeted to achieve a 25% relative reduction in premature mortality from cardiovascular disease, cancer, diabetes, or chronic respiratory diseases while targeting a reduction in NCD risk factors such a 25% relative reduction in the prevalence of raised blood pressure. Thus, strengthening HLC utilisation has been identified as a priority area in reorganising PHC in Sri Lanka to achieve above NCD targets by 2025 [12].

To the best of our knowledge, factors associated with the underutilisation of HLCs by its target population have not been investigated yet. However, available literature highlighted that the underutilisation of HLCs might be linked with low publicity about HLCs and perceptions related to health and wellbeing [13]. Systematic reviews based on other countries suggest that lower socioeconomic status, male gender, younger age, negative attitudes regarding the outcome of screening, low self-assessed health score, ongoing frequent or recent consultation at a general practice, and less social support are important factors of underutilisation of the screening services [16,17]. Most of the previous studies were done in developed countries, and there is a dearth of research evidence from developing countries. Hence, the present study aimed to describe the underutilisation and determine the factors associated with the underutilisation of HLCs in Sri Lanka.

## Methods

### Study design and study population

A cross-sectional study in the Gampaha and Kalutara districts, the adjacent two districts to the capital district of Sri Lanka. The selection of these districts was due to the high service delivery and availability of resources compared with other districts. Data was collected from May to June 2019.

The study population was 35- to 65-year-old adults who lived in the selected districts for at least six months before the data collection period. Individuals who were already diagnosed with chronic noncommunicable conditions, all three risk conditions (diabetes, hypercholesterolaemia, hypertension), pregnant and postpartum women (six months) were excluded because they are not included in the target population of the HLCs.

### Sample and sampling method

The sample size (n = 1950) wascalculated based on a standard formula [18] (expected proportion of individuals in the population who utilised the HLCs (p) = 0.255 [19], acceptable degree of absolute precision (d) = 0.03, level of significance (5%) (Z_1-α/2)_ = 1.96_)_, was adjusted for cluster sampling (design effect = 2). Respondents were selected using a five-staged sampling method.

Stage 1-. The cluster in the primary sampling unit was the catchment area of the HLC. Ministry of Health has not defined the catchment areas for HLCs formally and expects it to cover the catchment areas of the primary health care institutes. Some regional health offices have defined catchment areas, but they are not uniform across the regions. Therefore, considering the need for consistency and feasibility, the Village Administrative divisions (named as the *Grama Niladari* (*GN)* divisions, which are the lowest administrative division in Sri Lanka) within five kilometres from the selected HLCs were considered as the catchment areas for this study. Thirty catchment areas (15 catchment areas from each district) were selected using the simple random sampling method.

Stage 2- The cluster in the secondary sampling unit was the Village Administrative (*GN)* divisions. The average number of *GN* divisions in a catchment area of a HLC in the two districts was 14. Five *GN* divisions from each catchment area were randomly selected.

Stage 3- All names of residential blocks or streets (depending on the division) in the selected division were listed alphabetically with the support of the Village Administrative Officer (*Grama Niladari*). Three blocks/streets in a selected *GN* division were selected using the simple random sampling method.

Stage 4- Four/five households were selected systematically from the selected household block as follows.

#### Identifying the starting point

A starting point from the selected residential block or street was selected randomly using the area map available at the *GN* office. The first household located at that starting point was visited. If there were no eligible individuals in that household, that household was excluded, and the adjacent household on the left side was selected. This was continued until the initiating point was identified.

#### Selecting the rest of the households

Once the initiation point was identified, the next three households (four in the third block) were selected using the systematic sampling method. For this, a sampling frame was prepared for each selected block/street considering the number of households located in that selected block/street. The sampling interval was calculated using the aforementioned sampling frame and the number of households that was needed to be visited in each block. If there was no one at the selected household at the time of data collection, the particular data enumerator managed to verify the presence of at least one eligible member in that household, and a message for an appointment was sent to that resident. The data enumerator visited three times before it was labelled as a non-response.

Stage 5- Once the data enumerator visited the household, all eligible participants were listed. An individual among the eligible participants present at the household during the data enumerator’s visit was selected, applying a simple random sampling method using the lottery method. If there was only one individual that matched the inclusion criteria in the household, that respondent was selected automatically.

### Data collection instrument and method

A conceptual framework developed by a prior qualitative study by us was used to develop the interviewer-administered questionnaire used in this study [20]. According to this framework, HLC utilisation is principally influenced by the client’s cognitive and psychological attributes, family and community characteristics, and services-related perceptions, along with medical and screening history.

- **HLC utilisation**
  The outcome measure was the self-reported attendance at HLCs. Data enumerators verified the response based on the availability of the HLC record book. A binary variable was created indicating the HLC attendance (yes = 1, no = 0).
- **Client’s cognitive and psychological attributes**
  The client’s cognitive and psychological attributes encompassed knowledge on HLCs (using 2 multiple choice questions on aim and target diseases and 3 best of five questions on target age, population, and functioning date of the HLC), self-health assessment (using 0–100 rating scale), perceived susceptibility to NCDs or risk conditions (using five mutually exclusive responses), perceived usefulness of screening (using five mutually exclusive responses), enthusiasm on screening (using four items with a 3 point Likert scale), and enthusiasm to initiate and maintain a healthy lifestyle (using four items with a binary scale (yes and no)).
  Depending on the gradient of the responses in the factors of perceived susceptibility to NCDs or risk conditions and perceived usefulness of screening, each response was categorised into positive and negative perceptions. Perceived susceptibility to NCDs or risk conditions was defined as accurately perceiving the vulnerability to acquiring a common CVD and the risk conditions in the future.
- **Client’s family and community characteristics**
  Client’s family and community characteristics included perceived family support for screening and a healthy lifestyle (using six items with a 3-point Likert scale), acceptance of negative gender-related norms on screening (using nine items with a 5-point Likert scale), acceptance of negative norms related to NCDs and screening (using seven items with a 5 point Likert scale), perceived community networking (using nine items binary scale) and perceived presence of peer support (using 4 mutually exclusive responses to measure each perceived availability of supportive discussions and motivations) for NCD prevention, screening and healthy lifestyle. Perceived presence of peer support was classified as presence and absence, considering the categorisation of the responses of the two attributes.
- **Clients’ services-related perceptions**
  We obtained information on perceived negativity on functioning (using 4 items with binary responses), perceived quality of services (using a 5-point Likert type scale), and perceived accessibility to HLCs (using a 5-point Likert type scale).
- **Client’s medical and screening history**
  Data on family and personal history of NCDs or intermediate-risk conditions (presence of diabetes, hypertensionor hyperlipidemia) (dichotomous no and yes responses) and history of previous screening experience for blood sugar and cholesterol (dichotomous no and yes responses) was obtained.

Three experienced public health academics in state universities assessed the questionnaire for face and content validity. The developed questionnaire was pretested in an adjacent district (Kurunegala). Ethical clearance for the study was obtained from the Ethics Review Committee of the Faculty of Medicine, University of Kelaniya, Sri Lanka (P/141/07/2018). The data enumerator explained the study purpose and the procedures to the selected participants and obtained written consent before the data collection.

### Data analysis

Awareness and prevalence levels were calculated using descriptive statistics. Initially, the bivariate analysis was conducted using the Fisher Exact test, Mann-Whitney U test and Chi-Square Statistics to examine statistically significant differences between the utilisation of HLCs and relevant variables. The variables that were found to be statistically significant by the bivariate analysis were selected for the multivariable regression analysis. Assumptions were checked before conducting the adjusted logistic regression analysis and there was no multicollinearity among variables. Logistic regression models were fitted to estimate adjusted odds ratios of the utilisation of HLCs with 95% CIs and p-values, Analysis was conducted separately for men and women to prevent potential bias and identify sex-specific variations. Data analyses were conducted using the Statistical Package for Social Sciences version 23.

## Results

### Response rate and characteristics

Of the total 1950 approached, 1727 individuals responded, accounting for a response rate of 88.6%. Of the respondents, 59.8% (n = 1033) were females. The mean age was 49.8 (SD = 8.73) years (Female 49.71 (SD = 8.59), Male 49.89 (SD = 8.92)). The majority of the female respondents were housewives (n = 788, 76.3%), and most of the male respondents were daily wagers (n = 212, 30.5%). The mean household income was LKR 38036. 5 (USD 118.34) (SD = LKR 33865.6 (USD 105.36)) with a median household income of LKR 30,000 (USD 93.34) (IQR = LKR 30,000 (USD 93.34)), ranged from LKR 0 to LKR 600,000 (USD 1866.73) (Table 1).

**Table 1:** Distribution of study participants according to selected socio-demographic and economic characteristics.

Characteristic (n = 1727)	Frequency	%
**Sex**		
Female	1033	59.8
Male	694	40.2
**Age (Yrs)** (Mean = 49.8 (SD = 8.73))		
35–44	557	32.2
45–54	592	34.3
55–65	578	33.5
**Religion**		
Buddhist	1547	89.6
Others (Roman Catholic, Hindu, Islam, Christian)	180	10.4
**Level of education**		
No formal education	10	0.6
Primary education (Grade 1–5)	74	4.3
Lower Secondary (Grade 6–9)	1018	58.9
Upper secondary (Grade 10–13)	57.5	33.3
Tertiary (Bachelor and above)	50	2.9
**Marital status**		
Married	1644	95.2
Single	58	3.4
Divorced	04	0.2
Widow	16	0.9
Separated	05	0.3
**Employment Category**		
Stay-home mother	788	45.6
Unemployed (able to work)	77	4.4
Retired (with pension)	70	4.1
Self-employer	229	13.3
Daily wager	240	13.9
Non-government worker	185	10.7
Government worker	138	8.0
**Monthly household income (LKR)** (mean = LKR 38036. 5 (SD = LKR 33865.6))		
0–15,000	376	21.8
15,001–30,000	509	29.5
30,001–40,000	305	17.6
40,001–55,000	212	12.3
55,001–600,000	325	18.8
**Number of children** (mean 1.95 = (SD = 1.12))		
0	157	9.1
1–2	1124	65.1
3–4	429	24.8
More than 5	17	1.0
**Type of family**		
Nuclear	1220	70.6
Extended	507	29.4
**Distance to the nearest health facility (km)** (mean = 1.76 km (SD = 1.19 km))		
0–1	704	40.8
1.1–2	572	33.1
2.1–3	258	14.9
More than 3.1	193	11.2
**The available mode of transport to visit HLC**		
By public transport	354	20.5
By a rented three-wheeler	182	10.5
By an own vehicle	652	37.8
By walking	539	31.2

### Prevalence of HLC utilisation

Only 42.03% (n = 726; 95% CI: 39.7–44.4) were aware of the existence of an HLC in their locality or of its services. Of the total 1727 participants, only 195 (11.3%; 95% CI: 9.8–12.8) had utilised HLCs and the utilisation rate was higher among females (n = 165; 15.9%; 95% CI: 13.7–18.2) compared to males (n = 30; 4.3%; 95% CI: 2.81–5.8).

### Factors associated with HLC utilisation

#### Bivariate analysis (Table 2)

**Table 2:** Distribution of male and female clients according to probable factors associated with utilisation of HLCs: Results of bivariate analysis.

Factor	Female utilisation		Male utilisation		Total population utilisation	
	% (95% CI)	p-value	% (95% CI)	p-value	% (95% CI)	p-value
**Socio-demographic and economic characteristics**						
**Age groups (Yrs)**						
35–44	16.30 (12.40–20.30)	0.817^#^	0.90 (-0.03–2.17)	0.011^#^	10.2 (7.71–12.8)	0.532^#^
45–54	16.60 (12.73–20.51)		5.90 (2.88–8.93)		12.3 (9.67–14.9)	
55–65	15.0 (11.20–18.80)		5.90 (2.88–8.93)		11.2 (8.66–13.8)	
**Sex**						
Female	-	-	-	-	16.0 (13.74–18.21)	<0.001^#^
Male	-	-	-	-	4.3 (2.81–5.84)	
**Religion**						
Buddhism	16.63 (14.23–19.03)	0.090^#^	4.35 (2.74–5.96)	0.925^#^	11.7 (10.10–13.30)	0.116 ^#^
Non-Buddhist	10.28 (4.43–16.13)		4.11 (0.55–8.77)		7.78 (3.83–11.73)	
**Marital status**						
Married	16.35 (14.03–18.66)	0.589^##^	4.10 (2.58–5.61)	0.534^##^	11.4 (9.90–12.98)	0.802^##^
Single	11.54 (-1.62–24.70)		9.38 (-1.30–20.05)		10.3 (2.27–18.42)	
Divorced	N/A		N/A		N/A	
Widowed	7.14 (-8.29–22.57)		N/A		6.3 (7.07–19.57)	
Separated	N/A		N/A		N/A	
**Educational level**						
No formal education	14.29 (-20.67–49.24)	0.851^#^	N/A	0.784^#^	10.0 (-0.13–0.33)	0.960^#^
Primary	20.45 (8.05–32.86)		N/A		12.2 (4.54–19.79)	
Low secondary	16.33 (13.37–19.30)		4.31 (2.35–6.26)		11.4 (9.44–13.35)	
Upper secondary	15.21 (11.46–18.97)		5.00 (2.10–7.90)		11.3 (8.71–13.90)	
Above upper secondary	11.11 (-1.56–23.78)		4.35 (-4.67–13.36)		8.0 (0.21–15.79)	
**Occupation category**						
Stay-home mothers	16.88 (14.26–19.50)	0.762^#^	N/A	0.097^#^	16.9 (14.26–19.50)	<0.001^#^
Unemployed	22.22 (-11.67–56.12)		10.29 (2.88–17.70)		11.7 (4.35–19.03)	
Retired	11.76 (-5.31–28.84)		5.66 (-0.77–12.09)		7.1 (0.96–13.33)	
Self-employed	14.61 (7.12–22.09)		2.14 (-0.29–4.57)		7.0 (3.66–10.31)	
Daily wager	14.29 (0.47–28.10)		3.77 (1.19–6.36)		5.0 (2.22–7.78)	
Non-government	9.09 (0.25–17.93)		2.84 (0.06–5.61)		4.3 (1.37–7.28)	
Government	12.07 (3.43–20.71)		6.25 (0.83–11.67)		8.7 (3.94–13.46)	
**Type of family**						
Nuclear	17.96 (15.15–20.76)	0.008^#^	4.23 (2.46–6.01)	0.855^#^	12.4 (10.53–14.23)	0.027^#^
Extended	11.33 (7.77–14.88)		4.55 (1.62–7.47)		8.7 (6.22–11.14)	
**The available mode of transport to travel to HLC**						
Public transport	13.49 (9.25–17.74)	0.002^#^	4.90 (0.64–9.16)	0.454^#^	11.0 (7.74–14.29)	<0.001^#^
Hired three-wheeler	8.53 (3.64–13.41)		N/A		6.0 (2.55–9.54)	
Own vehicle	14.17 (9.79–18.55)		4.69 (2.62–6.76)		8.3 (6.16–10.40)	
Walking	20.99 (17.00–24.97)		4.48 (0.93–8.02)		16.9 (13.71–20.06)	
**Number of children**						
0	10.71 (3.96–17.47)	0.160^#^	6.85 (0.92–12.78)	0.538^#^	8.9 (4.41–13.42)	0.076^#^
1–2	15.05 (12.31–17.78)		3.65 (1.94–5.36)		10.3 (8.54–12.10)	
3–4	19.78 (15.07–24.50)		5.30 (1.68–8.91)		14.7 (11.32–18.05)	
More than 5	15.38 (-7.31–38.08)		N/A		11.8 (-5.31–28.84)	
**Distance from home to nearest health facility (km)**						
0–1	18.72 (14.98–22.46)	0.053^#^	6.03 (3.23–8.82)	0.317^#^	13.6 (11.10–16.18)	0.017^#^
1.1–2	16.52 (12.62–20.43)		3.17 (0.84–5.49)		11.4 (8.75–13.97)	
2.1–3	10.67 (5.67–15.66)		3.70 (0.08–7.32)		7.8 (4.47–11.04)	
More than 3.1	10.91 (4.99–16.83)		2.41 (-0.96–5.78)		7.3 (3.56–10.95)	
**Household income category (LKR per month)**						
0–15,000	13.24 (9.30–17.19)	0.009^#^	5.62 (0.74–10.50)	0.661^#^	11.4 (8.20–14.67)	0.015^#^
15,001–30,000	21.38 (16.75–26.02)		4.88 (1.90–7.85)		14.7 (11.65–17.82)	
30,001–40,000	17.34 (11.64–23.04)		2.27 (-0.30–4.85)		10.8 (7.31–14.33)	
40,001–55,000	15.13 (8.59–21.66)		3.23 (-0.43–6.88)		9.9 (5.85–13.96)	
55,001–600,000	9.33 (4.62–14.04)		5.14 (1.84–8.45)		7.1 (4.27–9.88)	
**Medical and screening history**						
**Family history of NCDs or a risk factor**						
Yes	18.17 (15.04–21.29)	0.027^#^	4.82 (2.57–7.06)	0.516^#^	13.16 (11.00–15.33)	0.007^#^
No	13.06 (9.92–16.21)		3.81 (1.77–5.86)		9.04 (7.03–11.06)	
**Personnel history of NCDs or a risk factor**						
No	16.29 (13.97–18.61)	0.248^#^	4.27 (2.75–5.80)	0.652^#^	11.36 (9.83–12.89)	0.667^#^
Yes	10.53 (2.31–18.74)		6.67 (-7.63–20.97)		9.72 (2.71–16.73)	
**Experience with either diabetes or cholesterol screening**						
No	6.62 (2.61–10.63)	<0.001^#^	2.35 (0.05–4.65)	0.213^#^	4.36 (2.12–6.61)	<0.001^#^
Yes	18.28 (15.59–20.98)		4.52 (2.67–6.37)		13.0 (11.2–14.9)	
**Cognitive and psychological attributes**						
**Perceived susceptibility to NCDs**						
Negative	3.07 (1.63–4.51)	<0.001^#^	0.67 (-0.09–1.42)	<0.001^#^	1.99 (1.13–2.85)	<0.001^#^
Positive	30.90 (26.74–35.05)		11.11 (7.13–15.09)		24.24 (21.11–27.37)	
**Perceived usefulness on screening**						
Negative	1.95 (0.90–3.01)	<0.001^#^	1.17 (0.24–2.10)	<0.001^#^	1.61 (0.89–2.33)	<0.001^#^
Positive	41.42 (36.35–46.48)		13.33 (8.32–18.35)		32.18 (28.25–36.10)	
**Knowledge on HLCs**	15.62^”*mean (95%CI)*”^ (14.65–16.58)	<0.001^###^	15.23^”*mean (95%CI)*”^ (12.89–17.58)	<0.001^###^	15.56^”*mean (95%CI)*”^ (14.68–16.44)	<0.001^###^
**Self-assessed health score**	68.48^”*mean (95%CI)*”^ (65.59–71.38)	0.180^###^	66.00^”*mean (95%CI)*”^ (60.36–71.64)	0.007^###^	68.10^”*mean (95%CI)*”^ (65.51–70.69)	0.005^###^
**Enthusiasm on screening**	33.48^”*mean (95%CI)*”^ (32.50–34.47)	<0.001^###^	33.50^”*mean (95%CI)*”^ (30.76–36.24)	<0.001^###^	33.49^”*mean (95%CI)*”^ (32.57–34.41)	<0.001^###^
**Enthusiasm for a healthy lifestyle**	19.82^”*mean (95%CI)*”^ (18.37–21.26)	<0.001^###^	20.67^”*mean (95%CI)*”^ (17.14–24.19)	<0.001^###^	19.95^”*mean (95%CI)*”^ (18.62–21.27)	<0.001^###^
**Family and community characteristics**						
**Perceived presence of peer support**						
Yes	23.01 (18.68–27.35)	<0.001^#^	6.10 (2.86–9.34)	0.125^#^	16.8 (13.73–19.84)	<0.001^#^
No	12.13 (9.64–14.61)		3.53 (1.88–5.19)		8.5 (6.91–10.15)	
**Acceptance of negative gender-related norms**	58.78^”*mean (95%CI)*”^ (56.54–60.91)	0.08^###^	59.83^”*mean (95%CI)*”^ (54.52–65.15)	0.155^###^	58.89^”*mean (95%CI)*”^ (56.89–60.90)	0.003^###^
**Acceptance of negative norms on NCDs and screening**	46.27^”*mean (95%CI)*”^ (44.29–48.25)	0.57^###^	48.33^”*mean (95%CI)*”^ (44.37–52.29)	0.797^###^	46.59^”*mean (95%CI)*”^ (44.82–48.36)	0.981^###^
**Perceived family support**	32.69^”*mean (95%CI)*”^ (31.39–34.01)	<0.001^###^	35.33^”*mean (95%CI)*”^ (31.69–38.96)	<0.001^###^	33.10^”*mean (95%CI)*”^ (31.87–34.34)	<0.001^###^
**Perceived community networking**	20.15^”*mean (95%CI)*”^ (18.57–21.73)	<0.001^###^	22.00^”*mean (95%CI)*”^ (17.31–26.69)	0.017^###^	20.44^”*mean (95%CI)*”^ (18.93–21.94)	<0.001^###^
**Services related perceptions**						
**Perceived accessibility to HLCs**						
Very high	19.44 (14.52–24.36)	0.033^#^	5.08 (-0.69–10.86)	0.001^#^	16.7 (12.55–20.89)	<0.001^#^
High	11.22 (7.70–14.74)		11.76 (3.91–19.62)		11.3 (8.12–14.52)	
Moderate	19.55 (14.75–24.35)		6.09 (2.97–9.20)		13.3 (10.31–16.31)	
Low	14.36 (9.30–19.42)		1.59 (0.2–2.98)		6.4 (4.23–8.52)	
Very low	13.33 (-6.15–32.82)		N/A		5.3 (-0.0217–0.1270)	
**Perceived quality of services**						
Totally-unsatisfied	8.40 (3.58–13.21)	<0.001^#^	3.37 (-0.45–7.19)	0.317^#^	6.4 (3.11–9.61)	<0.001^#^
Unsatisfied	9.89 (6.33–13.45)		2.75 (0.35–5.14)		7.0 (4.67–9.39)	
Neutral	19.18 (15.12–23.24)		4.07 (1.58–6.55)		13.1 (10.41–15.78)	
Satisfied	22.05 (16.91–27.18)		7.06 (3.17–10.95)		16.0 (12.53–19.54	
Totally satisfied	10.00 (-12.62–32.62)		N/A		5.9 (-0.0659–0.1835	
**Perceived negativity on functioning**	25.82^”*mean (95%CI)*”^ (24.57–27.07)	0.204^###^	24.33^”*mean (95%CI)*”^ (20.58–28.09)	0.560^###^	25.59”^*mean (95%CI)*”^ (24.40–26.78)	0.308^###^

^#^ Chi square test,

^##^ Fishers exact test,

^###^ Mann Whitney U test, Significance level p<0.05.

HLC utilisation was associated with most of the sociodemographic and economic characteristics: sex (p < 0.001), occupation category (p < 0.001), type of family (p = 0.027), available transport method to travel to HLC (p < 0.001), distance from home to nearest HLC (p = 0.017), status of having a personal income source (p < 0.001), and household income category (p = 0.015). Most of the users were from the female gender (n = 165, 15.9%; 95% CI: 13.7–18.2, p < 0.001) and were housewives (n = 133, 16.9%; 95% CI: 14.3–19.5, p < 0.001) compared to other occupational categories. Users were commonly belonged to nuclear families (n = 151, 12.4%; 95% CI: 10.5–14.2, p = 0.027). Walking was the preferred transport method (n = 91, 16.9%; 95% CI: 13.7–20.1, p < 0.001) and HLC usage was high when the distance to the HLC was less than 1 km from home (n = 96, 13.6%; 95% CI: 11.1–16.2, p = 0.017) compared to respectively other available transport methods and other distance categories. Use was high when there was no individual income source (n = 142, 16.4%; 95% CI: 13.9–18.9, p < 0.001). Users commonly belonged to the LKR 15,001–30,000 household income category (n = 75, 14.7%; 95% CI: 11.7–17.8, p = 0.015), the second-lowest household income category compared to other household income categories.

Among the medical and screening history variables, the status of family history regarding NCDs (p = 0.007) and lifetime experience of undergoing either cholesterol or diabetes screening (p < 0.001) was significantly associated with utilising HLCs. Users commonly had a positive family history of selected NCDs and risk factors (n = 124, 13.2%; 95% CI: 11.0–15.3, p = 0.007) and a lifetime experience of either cholesterol or diabetes screening (n = 167, 13.0%, 95% CI: 11.2–14.9, p < 0.001).

Utilisation was associated with each of the cognitive and psychological attributes. Users were aware of the existence or services of HLCs, (n = 195, 26.9%; 95% CI: 23.6–30.1, p < 0.001). Users commonly reported having positive perceptions on susceptibility to NCDs (n = 175,24.2%;95% CI: 21.1–27.4, p < 0.001), positive perceptions on the usefulness of undergoing screening (n = 176, 32.2%; 95% CI: 28.3–36.1, p < 0.001), higher mean knowledge (15.6 (SD = 6.26), Median = 17.0 (IQR = 9), Min = 0 Max = 25, p < 0.001), higher mean enthusiasm level on screening (33.5, (SD = 6.51), Median = 35.0 (IQR = 10), Min = 0 Max = 40, p < 0.001) and higher mean enthusiasm on healthy life (19.9, (SD = 9.39), Median = 20.0 (IQR = 20), Min = 0 Max = 40, p < 0.001) than non-users. Users had a lower mean score for self-health assessment which requested them to give a score to their health from a range of 0 to 100 (68.1 (SD = 18.3), Min = 0 Max = 100, p = 0.005)), than non-users.

Out of the family and community characteristics, all were significantly associated with utilisation except “acceptance of negative norms on NCDs and screening” (p = 0.981). Users reported a lower mean acceptance of negative gender-related norms (58.9 (SD = 14.2), Median = 60.0 (IQR = 20), Min = 0 Max = 90, p = 0.003)) than non-users.

Among the services-related perceptions, all variables were significantly associated with utilising HLCs except perceived negativity on functioning (p = 0.308). Users mostly reported having very high perceived accessibility to HLCs (n = 52, 16.7%, 95% CI: 12.6–20.9, p < 0.001) and a satisfied perception of the quality of the services (n = 68, 95% CI: 12.5–19.5, p < 0.001) compared with other perception categories in both variables.

#### Multivariable analysis (Table 3)

**Table 3:** Results of logistic regression analysis using utilisation status as the dependent variable for statically significant factors in bivariate analyses.

Variable	Female		Male		Total population	
	AOR (95% CI)	p value	AOR (95% CI)	p value	AOR (95% CI)	p value
**Socio-demographic and economic factors**						
**Sex**						
Female*	-	-	-	-	-	0.002
Male	-	-	-	-	0.18 (0.05–0.52)	
**Occupation category**						
Stay-home mother*	-	0.300	-	-	-	0.412
Unemployed **	0.88 (0.02–33.27)	0.943	-	0.470	3.29 (0.54–20.28)	0.199
Retired	0.03 (0.00–1.45)	0.076	0.001 (0.00–0.88)	0.046	0.29 (0.04–2.47)	0.262
Self-employed	1.04 (0.23–4.78)	0.962	0.000 (0.00–35.33)	0.148	0.85 (0.24–3.05)	0.805
Daily wager	3.45 (0.51–23.54)	0.206	0.08 (0.00–51.66)	0.446	1.98 (0.50-)	0.326
Non-government	0.17 (0.02–1.74)	0.136	0.05 (0.00–56.71)	0.398	0.47 (0.10–2.19)	0.340
Government	1.11 (0.19–6.47)	0.911	0.00 (0.00–5.18)	0.097	1.09 (0.28–4.27)	0.900
**Type of family**						
Nuclear *	-	0.035	-	0.644	-	0.021
Extended	0.39 (0.16–0.94)		0.50 (0.03–9.37)		0.43 (0.21–0.88)	
**The available mode of transport to visit HLC**						
Public transport*	-	0.971	-	0.632	-	0.821
Hired three-wheeler	0.69 (0.15–3.13)	0.631	0.00 (0.00)	0.998	0.52 (0.14–2.03)	0.349
Own vehicle	0.91 (0.30–2.76)	0.870	52.99 (0.12–26716.96)	0.211	0.87 (0.34–2.19)	0.761
By walking	0.93 (0.33–2.67)	0.896	46.50 (0.02–89748.38)	0.320	0.79 (0.31–1.99)	0.610
**Distance to nearest health facility (km)**						
0–1*	-	0.025	-	0.253	-	0.004
1.1–2	0.31 (0.12–0.77)	0.011	0.01 (0.00–1.18)	0.058	0.29 (0.14–0.63)	0.002
2.1–3	0.59 (0.14–2.56)	0.480	0.02 (0.00–13.27)	0.241	0.46 (0.15–1.39)	0.169
More than 3.1	0.14 (0.03–0.61)	0.009	0.00 (0.00)	0.110	0.14 (0.04–0.53)	0.004
**Household income category (LKR per month)**						
0–15,000*	-	0.033	-	0.346	-	0.013
15,001–30,000	3.92 (1.41–10.93)	0.009	340.69 (0.44–262174.87)	0.085	3.69 (1.53–8.89)	0.004
30,001–40,000	3.29 (1.04–10.37)	0.042	26.64 (0.03–23994.07)	0.344	2.84 (1.02–7.94)	0.046
40,001–55,000	1.74 (0.45–6.66)	0.420	0.02 (0.00–157.29)	0.380	1.15 (0.37–3.63)	0.807
55,001–600,000	0.68 (0.17–2.81)	0.595	114.20 (0.02–821508.06)	0.296	0.99 (0.30–3.24)	0.987
**Medical and screening history**						
**NCD family history**						
No*	-	0.562	-	0.067	-	0.164
yes	0.79 (0.37–1.71)		0.012 (0.00–1.37)		0.63 (0.33–1.21)	
**Experience with either blood sugar or cholesterol screening**						
No *	-	0.269	-	0.671	-	0.113
Yes	0.39 (0.08–2.05)		0.37 (0.00–34.37)		0.366 (0.11–1.27)	
**Cognitive and psychological attributes**						
**Perceived usefulness on screening**						
Negative perception*	-	<0.001	-	0.029	-	<0.001
Positive perception	14.89 (5.43–40.81)		933.52 (2.01–434253.29)		10.15 (4.40–23.38)	
**Knowledge on HLCs**	1.32 (1.24–1.41)	<0.001	2.70 (1.315.56)	0.007	1.31 (1.24–1.38)	<0.001
**Self-assessed health score**	0.97 (0.95–0.99)	0.015	0.95 (0.86–1.05)	0.335	0.97 (0.95–0.99)	0.001
**Perceived susceptibility to NCDs**						
Negative perception*	-		-		-	
Positive perception	5.03 (1.72–14.65)	0.003	310.95 (3.81–25368.32)	0.011	6.78 (2.79–16.42)	<0.001
**Enthusiasm on screening**	1.10 (1.044–1.17)	0.002	1.144 (0.92–1.39)	0.230	1.09 (1.04–1.14)	<0.001
**Enthusiasm for a healthy lifestyle**	1.01 (0.97–1.06)	0.518	0.93 (0.79–1.11)	0.428	0.99 (0.97–1.03)	0.959
**Family and community characteristics**						
**Acceptance of negative norms related to gender**	1.03 (1.00–1.06)	0.040	1.06 (0.95–1.19)	0.316	1.02 (0.99–1.04)	0.064
**Perceived family support**	1.15 (1.10–1.21)	<0.001	1.35 (1.047–1.727)	0.020	1.14 (1.09–1.19)	<0.001
**Perceived community networking**	1.11 (1.06–1.16)	<0.001	1.09 (0.92–1.29)	0.343	1.08 (1.04–1.12)	<0.001
**Perceived presence of peer support**						
Yes						
No	5.79 (2.36–14.17)	<0.001	0.044 (0.00–2.03)	0.108	3.12 (1.53–6.34)	0.002
**Services related perceptions**						
**Perceived accessibility to HLCs**						
Very high *		0.001		0.211		0.001
High	0.28 (0.09–0.85)	0.025	223.11 (0.24–210360.59)	0.122	0.44 (0.17–1.16)	0.096
Moderate	1.12 (0.37–3.38)	0.848	1.65 (0.00–838.61)	0.876	0.96 (0.37–2.48)	0.926
Low	0.10 (0.03–0.39)	0.001	0.09 (0.00–17.65)	0.368	0.12 (0.04–0.36)	<0.001
Very low	2.39 (0.12–48.49)	0.569	0.00 (0.00)	0.997	0.50 (0.04–5.744)	0.581
**Perceived quality of services**						
Totally-unsatisfied*		0.012		0.698		0.015
Unsatisfied	0.734 (0.13–4.10)	0.727	0.17 (0.00–154.17)	0.614	0.59 (0.15–2.36)	0.460
Neutral	3.52 (0.70–17.58)	0.126	10.89 (0.04–3383.15)	0.415	2.27 (0.66–7.799)	0.192
Satisfied	3.37 (0.65–17.47)	0.148	0.67 (0.01–86.31)	0.874	2.12 (0.61–7.45)	0.240
Totally satisfied	0.06 (0.00–3.59)	0.176	0.00 (0.00)	0.999	0.03 (0.00–2.60)	0.124
Constant	0.00	<0.001	0.00	0.032	0.00	<0.001

*Reference category

**Reference category of males for occupation category.

AOR: Adjusted Odds Ratio.

After mutual adjustment for all characteristics categories, among sociodemographic and economic characteristics, only sex, type of family, distance to the nearest HLC and household income category were significantly associated with HLC utilisation. Among cognitive and psychological attributes, the association between perceived usefulness on screening, perceived susceptibility to NCDs, knowledge on HLCs, self-assessed health score and enthusiasm on screening with HLC utilisation remained after multivariable adjustment while enthusiasm for a healthy life did not show an association. Under family and community factors, perceived family support, perceived community networking and perceived peer support remained as predictors of HLC utilisation while acceptance of negative gender-related norms was not related to HLC utilisation after multiple adjustments. Under services-related perceptions, perceived accessibility and perceived quality of services remained associated with HLC utilisation. Medical and screening history variables did not predict utilisation behaviour in multivariable analysis.

As there was a significant difference between males and females, a comprehensive subgroup analysis was done to understand the specific factors influencing the underutilization among the two gender groups.

In females, belonging to an extended family (OR = 0.39 (95% CI: 0.16–0.94, p = 0.035) was associated with decreased odds of utilising HLCs compared with those belonging to a nuclear family. Females had 0.31 (95% CI: 0.12–0.77, p = 0.011) and 0.14 (95% CI: 0.03–0.61, p = 0.009) times lower odds of utilising HLCs if they resided 1-2km and more than 3km distance categories compared with females resided in less than 1km distance. Females from the second (15,001–30,000 LKR) and third (30,001–40,000 LKR) lowest household income categories had a 3.92 (95% CI: 1.41–10.93, p = 0.009) and 3.29 (95% CI: 1.04–10.37, p = 0.042) times higher odds of become a user compared with females belong to the lowest household income category (<15,000 LKR).

Among cognitive and psychological attributes, positive perceptions on the usefulness of screening (OR = 14.89 (95% CI: 5.43–40.8), p < 0.001), positive perceptions on susceptibility to NCDs (OR = 5.03 (95% CI: 1.72–14.7), p = 0.003), increment in knowledge (OR = 1.32 (95% CI: 1.24–1.41), p < 0.001), and increment in enthusiasm on screening (OR = 1.10 (95% CI: 1.04–1.17), p = 0.002) presented as predictors of HLC utilisation. An increment in the self-health score (OR = 0.97 (95% CI: 0.95–0.99), p = 0.015) was related to lower utilisation. Under family and community factors, female users were found to be increased by 1.03 (95% CI: 1.00–1.06, p = 0.040) times with a unit increment in acceptance on norms related to gender, 1.15 (95% CI: 1.10–1.21, p < 0.001) times with a unit increment in perceived family support, 1.11 (95% CI: 1.06–1.16, p < 0.001) times with a unit increment in perceived community networking. Females with perceived peer support had 5.79 (95% CI: 2.36–14.17, p < 0.001) times higher odds of becoming a HLC user compared with their counterparts. Under services-related perceptions, females who perceived accessibility to HLC as high or low respectively had a 0.28 (95% CI: 0.09–0.85, p = 0.025) and 0.10 (95% CI: 0.03–0.39, p = 0.001) times lower odds of become a user compared with females who perceived accessibility to HLC is very high.

In males, the retired occupation category was associated with a decreased odds of (OR = 0.001 (95% CI: 0.00–0.88), p = 0.046) utilising HLCs compared with unemployed males. Positive perceptions on the usefulness on screening (OR = 310.95 (95% CI: 3.81–25368.32), p = 0.029), positive perceptions on susceptibility to NCDs (OR = 933.52 (95% CI: 2.01–434253.29), p = 0.011), increment in knowledge on HLCs (OR = 2.70 (95% CI: 1.31–5.56), p = 0.007) and increment in perceived family support (OR = 1.35 (95% CI: 1.05–1.73), p = 0.020) were the only other predictors of male HLC utilisation.

## Discussion

### Summary of findings

This study aimed to describe the underutilisation of HLCs and determine the factors associated with the utilisation of HLCs in Sri Lanka. The awareness about the existence of HLCs in the study sample was 42% (n = 726, 95% CI: 39.7–44.4). The prevalence of utilising HLCs was 11.3% (n = 195, 95% CI: 9.80–12.8) and females (n = 165; 16.0%; 95% CI: 13.74–18.21) had a higher utilisation compared to males (n = 30; 4.3%; 95% CI: 2.81–5.84). After multivariable analysis, 14 factors were significantly associated with the utilisation of HLCs. The most influential factor was having a positive perception on the usefulness of screening (OR = 10.2, 95% CI: 4.04–23.4). In addition, the odds of utilising HLCs had increased by respectively 6.78 (95% CI: 2.79–16.42) and 3.12 (95% CI: 1.54–6.34) times if a respondent thought s/he is susceptible to NCDs and if s/he perceived presence of peer support for screening and a healthy lifestyle. Other significant predictors that improve HLC utilisation were the second and third lowest household income categories of 15,001–30,000 LKR (OR = 3.69, 95% CI: 1.53–8.89) and 30,001–40,000 LKR (OR = 2.84, 95% CI: 1.02–7.94), higher level of knowledge on HLCs (OR = 1.31, 95% CI: 1.24–1.38), higher enthusiasm on screening (OR = 1.09, 95% CI: 1.04–1.14), higher perceived family support (OR = 1.14, 95% CI: 1.09–1.19) and higher perceived community networking (OR = 1.08, 95% CI: 1.04–1.12). Male sex (OR = 0.18, 95% CI: 0.05–0.52), belonging to an extended family (OR = 0.43, 95% CI: 0.21–0.88), resided within 1–2 km (OR = 0.29, 95% CI: 0.14–0.63) or more than 3 km (OR = 0.14, 95% CI: 0.04–0.53) of the HLC, having a higher self-assessed health score (OR = 0.97, 95% CI: 0.95–0.99) and low perceived accessibility to HLCs (OR = 0.12, 95% CI: 0.04–0.36) were significantly reduced the HLC utilisation. Similarities between males and females were observed with regard to the type of perception on susceptibility to NCDs and the usefulness on screening, knowledge on HLCs, and perceived family support. The first two strong positive predictors of HLC utilisation among both genders were positive perceptions on the usefulness on screening and positive perceptions on susceptibility to NCDs.

### Implications and comparison with other study findings

Our findings align with previous global studies showing that males were less likely to undergo screening than females [16,17,21,22]. The female-to-male utilisation ratio of the current study was 5.5:1. This disproportionate ratio is higher than the country rates in 2018 (2.2:1) and 2019 (2.6:1)[23]. Reporting a high percentage of female users is compatible with the consecutive national reports for the past ten years [9]. We found that this is due to relatively high health-seeking behaviour among females compared to males, which was also consistent with the global literature [16,17]. The commonest reason that influences health health-seeking behaviour of the males was masculine perceptions [24–27], which was also consistent with our prior qualitative study done with the same study population [20]. Our qualitative findings indicated that gender stereotyping defines boundaries for men not to opt for healthy choices common to both males and females. This might be due to social stigma and negative peer influence associated with deviation from gender norms [24]. Thus, males were unprepared to utilise screening and lifestyle modification services compared to females, a well-established risk factor for increased NCD risk, leading to increased premature mortality and reduced life expectancy among them [16]. Previous literature indicates that the opportunity to participate without gender bias is a key measure of the success of community screening programmes [28]. Thus, current study findings imply the need for more effective promotional campaigns such as social marketing, social media, or mass media targeted at males that address all the relevant norms and modifications to the current services to accommodate male clients [29].

We have found that users were commonly from the second lowest household income category. Reporting a greater number of users with a lower household income is consistent with previous studies in developing countries namely India [30], Malaysia [31], and Nigeria [32]. In contrast to this, users in developed countries were commonly from higher income categories as evidenced by previous systematic reviews [16,17]. However, our study has also found that there was an access issue for the first household income category people (lowest level of income) to the HLC because the least number of users were reported from this category compared with other categories. According to our previous qualitative study on the same topic and the population, the daily wagers do not give up their daily income just participate in a screening session conducted on a weekday morning, especially when they don’t have any symptoms [20]. Even though, multivariable analysis have confirmed that household income category is a significant predictor for total population and females. Our findings implied that HLCs are serving impoverished groups by being affordable to them. Thus, in a way, our study provides evidence that the service aim of the Ministry of Health is achieved with some lowest levels of household income groups. However, at the same time our study also provides evidence for the fact that the service should be reorganized to cater for the lowest-income group of Sri Lanka.

Being a member of the nuclear family and living less than one kilometre from the HLC were significant predictors of female HLC utilisation, indicating that if immediate social and physical access-related barriers are overcome, they will utilise the HLC. However, none of the sociodemographic and economic characteristics was significantly associated with males except being retired from the occupation. This can be interpreted as evidence that occupation is a main barrier to utilisation for males, even when other barriers are absent [16]. This strongly highlights the need to change the service delivery structure, if to improve male participation.

None of the medical and disease history factors was significantly associated with HLC utilisation among both males and females in the multivariable analysis. It implies that the factors that were positive in the bivariate analysis were positive due to the effect of confounding by other variables that were included in the multivariable analysis. As a systematic review of global literature also reports them as neutral factors for self-motivation for screening, one may decide to ignore their importance [16]. However, as some of the primary studies have reported them as facilitators and persons with a family or medical history have a well-proven increased risk for NCDs, it is advisable not to ignore this factor in planning interventions and promotion strategies related to HLC utilisation [33].

We found having positive perceptions on susceptibility to NCDs and the usefulness on screening were the most influential predictors of HLC utilisation by both genders. Psychological models such as the health belief model, theory of reasoned action, and health action process approach also demonstrate how proactive perceptions on susceptibility guide individuals to change behaviour [34]. However, there was inconsistent evidence on perceived susceptibility to NCDs as a predictor of screening utilisation. Some literature showed higher perceived susceptibility as a more robust predictor [34] while other findings did not support that claim [35–37]. However, one intervention conducted in the United States reported that individuals utilised screening services more after their risk perception improved [38]. Thus, these findings highlight that the target population will not utilise HLCs if screening-specific perceptions are not positive.So, it is important to aim at developing positive perceptions without merely improving knowledge in future HLC promotions. This fact was highlighted in the global literature as well [2,5]. According to the sex-specific analysis, males had a higher chance of utilising HLCs if they had a positive perception of susceptibility to NCDs and the usefulness on screening, knowledge on HLCs, and enthusiasm on screening than females. These findings highlight important implications for Sri Lankan policymakers on HLC to design male-specific strategies to improve HLC utilisation among them. Based on our findings, such male-specific strategies should be designed to improve positive perceptions on susceptibility to NCDs and usefulness on screening, knowledge on HLCs and enthusiasm on screening and can range from male-sensitive educational interventions and video-based educational interventions to partner educational interventions [29,39].

A previous study conducted in Germany reports that males had higher odds of utilising screening services with high or intermediate social support compared to females [3]. In contrast, we found that the perceived presence of peer support was only associated with the total population and females. Global literature showed that social networks [40], social support [41], and neighbourhood social cohesion [42] as vital determinants of better health and health behaviour. These factors are conceptually similar to perceived community networking in our study which showed a higher HLC utilisation by individuals with higher perceived community networking than their counterparts. In the sex-specific analysis, this factor was also significantly associated with female HLC utilisation. These findings imply that families, peers and communities can play significant roles in improving attendance to screening programmes within communities as indicated by the two action areas in the Ottawa Charter for health promotion: Strengthening community action and developing a supportive environment for health [43]. Under strengthening community action, health professionals can empower different community segments to conduct the situational analysis of HLC utilisation, design and implement collective actions to address determinants of HLC utilisation within their communities and monitor the progress. Developing a supportive social environment is important to enlighten the community about the importance of screening, improve positive perceptions of susceptibility of NCDs, share knowledge about HLCs, provide peer support and for community networking. A supportive physical environment such as timeslots within existing community-based organisations is essential to provide avenues for sharing knowledge about HLCs and to improve enthusiasm on screening. These avenues will also benefit from improving peer support and community networking for HLC utilisation [20,43]. Thus, the above findings highlight a need to consider these factors apart from cognitive and psychological factors when developing interventions to improve HLC utilisation.

Previous studies highlighted that low screening utilisation was due to the masculine views of men [3,24]. Nevertheless, we only observed an increased odds of utilising HLCs by females with a unit increment in acceptance of negative gender norms. Social norms are a factor considered important in behaviour change theories, such as the theory of planned behaviour and the theory of reasoned action [34]. However, a meta-analysis showed that to date, subjective norms possessed only a medium-sized relationship to participating in screening [44]. In our study, acceptance of negative norms on NCDs and screening was not found to be associated with utilisation. This can be because there was a high degree of acceptance for these norms irrespective of the utilisation status among the study participants.

Perceived accessibility and quality of the services were significant predictors of HLC utilisation which was also reported by another local study [45]. Global literature showed that improving accessibility via flexible appointment times and conducting screening after office hours and on weekends would probably increase public participation, which is also applicable to the HLCs [46]. Our findings imply the need to improve the accessibility and quality of the services parallel to the increasing demand for HLCs. However, the implementation of such measures will be challenged by the availability of human resources and health financing issues in low and middle-income countries [35]. Literature shows that community-based intervention could improve public screening participation in low and middle-income countries [47]. Therefore, power can be delegated to local communities to promote the HLC, through improving family and peer support, and community networking. For this purpose, innovative community-based promotions that are designed on identified predictors can be used to market unique services like health education sessions, lifestyle modification sessions, and follow-ups.

### Strengthens and limitations

There were several strengths of this study. (1) first community-based study about potential factors associated with HLC utilisation in Sri Lanka (2) testing factors related to five-variable categories exclusively identified by a prior qualitative study (3) logistic regression model to account for possible confounders of HLC utilisation. The external validity was ensured by obtaining a representative sample using an appropriate probability sampling method. The, sample size was decided using a standard formula to ensure an adequate sample size. Adequate coverage of the study sample was achieved by using measures to reduce non-response. Non-response was avoided by training enumerators to effectively explain the study’s general objective and importance. In the absence of inhabitants in the selected households, the houses were visited at least three times before classifying them as “non-respondent.” Thus, results can be generalized to the study population.

However, our study design was cross-sectional and thus could not establish causal relationships like in prospective studies. Measures were taken to account for identified bias to ensure internal validity of data. As the data were collected through an IAQ, interviewer bias could have occurred. Therefore, enumerators were trained on building rapport with the interviewees, not showing judgmental reactions to the responses, and not giving cues on expected answers. There could be recall bias for HLC clients because certain questions were asked about the client’s perceptions or experiences before they visited the HLC. Therefore, the reported answers for perceived susceptibility to NCDs, perceived usefulness of screening, enthusiasm on screening and enthusiasm on healthy lifestyle could be overestimated responses. HLC client’s could report a higher knowledge about HLCs due to their visits to the HLC. However, it was assumed that this effect is trivial as improving the knowledge on HLCs is not targeted in the health education session. There was a possibility of receiving social desirability answers for items assessing perceived family support, community networking and peer support and services related perceptions prior to their HLC visit. It was assumed that there might be no or minimal effect from HLC visit on these variables for clients as there is no systematic involvement to change these variables from HLC. However, enumerators were trained mainly on the method of administering all above types of questions to minimize bias. Moreover, there can be response bias in getting the answer for the household income, because we used a direct answer question for that variable. It is a limitation of our study. There were statistical concerns as the actual strength of the association between tested factors and male utilisation was inconclusive due to the large standard error in odds ratios caused by the lower number of male users.

### Conclusion and recommendations

Awareness and utilisation of the freely available HLCs are low among the target population in Sri Lanka. Multiple factors operating at different levels accounted for the underutilisation of HLCs. Therefore, HLC utilisation would not be improved by only improving knowledge on HLCs, because of the influence of personal attitudes along with wide community factors namely family support, community networking and peer support. Thus, interventions aiming to improve HLC utilisation should be complex and multifaceted designs addressing these factors. Moreover, according to the bivariate and multivariable analysis these predictors of HLC utilisation differed with gender, with men showing low utilisation. Hence, gender-sensitive innovative interventions will potentially improve HLC utilisation, including specific strategies focusing on men.

## Supporting information

S1 Data set (SAV)

S1 Questionnaire (DOCX)
