# Supplementary material for: Under-utilisation of noncommunicable disease screening and healthy lifestyle promotion centres: A cross-sectional study from Sri Lanka
Source: PLoS One. 2024 Apr 4;19(4):e0301510. doi: 10.1371/journal.pone.0301510 (PMC10994285; doi:10.1371/journal.pone.0301510)
Supplement: S1 Questionnaire — (DOCX) [file pone.0301510.s002.docx]

**Annexe 7**: **Interviewer Administered Questionnaire**

**S/no-**

There are several sections in this questionnaire, and the data collector should fill out the questionnaire after obtaining data from the participant.

| **Date** | **DS Division** | **Name of the HLC** | **GN division** | **Village** | **Area of residence** | **Name of the data collector** |
| --- | --- | --- | --- | --- | --- | --- |
|  |  |  |  |  |  |  |
| **GIS** | **Longitude** | *(enter in decimals)* | | | | |
|  | **Latitude** | *(enter in decimals)* | | | | |

**Section A: Socio-demographic and economic data**

| 1. | What is your age at last birthday?............... | 6.  7. | a.How many years did you completely attend school except your preschool years? ……………..  b.What is your highest educational qualification?........................................  What is your current occupation?....................................... |
| --- | --- | --- | --- |
| 2. | Tick the sex of the participant.   \| Female \| 1 \| \| --- \| --- \| \| Male \| 2 \| | 8. | How many family members are there in your house? …..  Mention whether the family is a nuclear or an extended   \| nuclear \| 1 \| \| --- \| --- \| \| extended \| 2 \| |
| 3. | What is your marital status?   \| Married \| 1 \| \| --- \| --- \| \| Unmarried \| 2 \| \| Divorced \| 3 \| \| Widowed \| 4 \| \| Separated \| 5 \| \| Living together \| 6 \| \| Can’t mention \| 88 \| | 9.  10.  11. | How many children do you have?...................  Can you state your monthly salary? ………………..  How many family members are doing some kind of an earning in your family?..............  Can you state their total monthly earnings of them? …………………. |
| 4. | What is your nationality?   \| Sinhala \| 1 \| \| --- \| --- \| \| Sri Lankan Tamil \| 2 \| \| Indian Tamil \| 3 \| \| Muslim \| 4 \| \| Burger \| 5 \| \| Other (Specify) \| 6 \| | 12. | What is the average distance from your home to the nearest PMCU/CD/BH/DH? ……………………… |
| 5. | What is your religion?   \| Buddhist \| 1 \| \| --- \| --- \| \| Roman Catholic \| 2 \| \| Hindu \| 3 \| \| Islam \| 4 \| \| Christian \| 5 \| | 13. | If you need to go to the nearest PMCU/CD/BH/DH what will be the preferred mode of transport that you use?   \| By public transport \| 1 \| \| --- \| --- \| \| By a rented three-wheeler \| 2 \| \| By an own vehicle \| 3 \| \| By walking \| 4 \| |

**Section B: Level of knowledge about HLCs**

| 14. | Have you ever heard about Healthy Lifestyle Centers?   \| Yes **Go to question 15** \| 1 \| \| --- \| --- \| \| No **Go to section 22** \| 0 \| | 20. | How did you know about HLC for the first time? ***(tick the most relevant answer)***   \| Health worker \| 1 \| \| --- \| --- \| \| Television/Radio/Newspapers \| 2 \| \| Relatives/friends/early participants \| 3 \| \| Invitation/Board/poster displayed at the health institution \| 4 \| \| Community health promotion programme \| 5 \| \| Other (Mention) \| 6 \| \| Don’t remember \| 7 \|   **If 1 is an answer, go to question 21** |
| --- | --- | --- | --- | --- | --- | --- | --- | --- | --- | --- | --- | --- | --- | --- | --- | --- | --- | --- | --- | --- | --- |
| 15. | What is a Healthy Lifestyle Center? ***(Can tick more than one answer)***   \| It is a screening service for NCD risk factors…2 marks \| 1 \| \| --- \| --- \| \| It provides lifestyle modification advice…2 marks \| 2 \| \| As a follow-up service to NCD risk…2 marks \| 3 \| \| Don’t remember…0 marks \| 4 \| \| No idea…0 marks \| 5 \| | 21. | What was the source of information you came to know via a health worker?   \| Home visit by a health worker \| 2 \| \| --- \| --- \| \| Health talk at a PMCU/CD/DH/BH \| 3 \| \| Health talk at OPD \| 4 \| \| When consulting a doctor for another disease/ (Message /referral from a medical officer) \| 5 \| \| Other (Mention) \| 6 \| \| Don’t remember \| 7 \| |
| 16. | What are the NCDs or risk conditions targeted by the HLC? ***(Can tick more than one answer)***   \| Diabetes…2 marks \| 1 \| \| --- \| --- \| \| Hypertension…2 marks \| 2 \| \| Hypercholesteremia …2 marks \| 3 \| \| Cancer…2 marks \| 4 \| \| Don’t remember…0 marks \| 5 \| \| No idea…0 marks \| 6 \| | 22.. | Healthy Lifestyle Centre is a clinic where you can measure your blood sugar and cholesterol. People who do not have NCDs will be invited. It provides lifestyle modifications and some centres have an exercise programme. This centre is in your village PMCU/CD/DH/BH. Have you heard about this centre?   \| No **… Go to section** **23** \| 0 \| \| --- \| --- \| \| Yes **…Go to question 20** \| 1 \| |
| 17. | Who is the target gender of HLCs?   \| Women…0 marks \| 1 \| \| --- \| --- \| \| Men…0 marks \| 2 \| \| Both…2 marks \| 3 \| \| Don’t remember…0 marks \| 4 \| \| No idea…0 marks \| 5 \| |  |  |
| 18.. | What is the targeted age group of HLCs?   \| 35-65 years …2 marks \| 1 \| \| --- \| --- \| \| Above 18 years …0 marks \| 2 \| \| Above 65 years …0 marks \| 3 \| \| Don’t remember…0 marks \| 4 \| \| No idea…0 marks \| 5 \| |  |  |
| 19. | Tell me the day when your area HLC is conducting?   \| Answer corrects ……7 marks \| 1 \| \| --- \| --- \| \| Answer incorrect ……0 marks \| 2 \| |  |  |

**Section C: Utilisation of HLCS**

| 23. | Have you visited HLC, at least once?   \| Yes…..**Go to question 24** \| 1 \| \| --- \| --- \| \| No…..**Go to question 27** \| 0 \|   ***Instructions: Verify from the HLC record book****.*  Date of the visit 1   \| Year \| Month \| Date \| \| --- \| --- \| --- \| \|  \|  \|  \| | 25. | What are the dietary habits you have changed up to now? **(*Can tick more than one).***   \| Have reduced rice portion per meal \| 1 \| \| --- \| --- \| \| Have reduced number of servings \| 2 \| \| Have reduced number of teaspoons of sugar \| 3 \| \| Have reduced sugar-dense food \| 4 \| \| Have reduced salt-dense food \| 5 \| \| Have reduced the quantity of salt per curry when cooking \| 6 \| \| Have reduced adding salt when cooking rice \| 7 \| \| Have reduced oily food \| 8 \| \| Have reduced the quantity of oil per curry when cooking \| 9 \| \| Have an increased number of vegetables consumed per day \| 10 \| \| Have an increased number of fruits consumed per day \| 11 \| \| Have reduced instant food consumption \| 12 \| \| Other (Specify)……………………. \| 13 \| |
| --- | --- | --- | --- | --- | --- | --- | --- | --- | --- | --- | --- | --- | --- | --- | --- | --- | --- | --- | --- | --- | --- | --- | --- | --- | --- | --- | --- | --- | --- | --- | --- | --- | --- | --- | --- | --- | --- | --- | --- |
| 24. | Currently, are you following advice given by the HLC at your home?   \| Dietary habits \| 1 \| \| --- \| --- \| \| Physical inactivity \| 2 \| \| Both \| 3 \|   ***If dietary habits are mentioned, go to question 25. If physical activity is mentioned go to question 26. If both are mentioned, go to both 25 and 26*** | 26. | What activities are you doing to increase physical activity? ***(Can tick more than one)***   \| Participating in the exercise programme at the HLC \| 1 \| \| --- \| --- \| \| Conducting exercises at home \| 2 \| \| Exercise on the ground or walking paths \| 3 \| \| Home garden \| 4 \| \| Increase the normal speed of day-to-day activities \| 5 \| \| Engaging in a sport \| 6 \| \| Other (Specify)…………………… \| 7 \| |

**Section D: Other factors associated with the utilisation of HLC**

***Instructions: Explain the below description to the respondent.***

**Different people have different perceptions of health, NCD risk and screening. These questions only aim to capture those perceptions. There are no correct and wrong answers. And by giving an answer you will be not measured, stigmatised and humiliated.**

***Instructions: Ask about the perception/situation before HLC utilisation from HLC clients***

| **27.** | **Self-assessed health score** | **29.** | **Perceived usefulness of screening** |
| --- | --- | --- | --- |
|  | In general, how would you rate your health?............  *(Use the laminated cards)*    100  50  0 |  | Do you need to check for blood sugar or blood cholesterol or blood pressure even without risk factors/symptoms? *(Use the laminated cards)*   \| No need to check unless we have symptoms or a disease \| 1 \| \| --- \| --- \| \| It’s pointless to continue if we know our status from an earlier test \| 2 \| \| Can check if a doctor tells \| 3 \| \| It's good if we check even without evident risk factors \| 4 \| \| It’s good to check for blood sugar as diabetes is common \| 5 \| |
| **28.** | **Perceived susceptibility to NCDs** |  | |
|  | What do you feel about your risk to NCD? *(Use the laminated cards)*   \| I don’t think I’m at risk \| 1 \| \| --- \| --- \| \| Unless a symptom appears I will not think of a risk \| 2 \| \| I have a family history so I’m concerned \| 3 \| \| I may be at risk \| 4 \| \| Though I have NCDs I don’t think I will get another NCD \| 5 \| |  |  |

30. Have you ever screened for high blood sugar or cholesterol?

| Yes……………1 | **If yes go to 30 (A) Section** |
| --- | --- |
| No…………….2 | **If no go to 30 (B) Section** |

***Instructions: Explain the below description to the respondent.***

**Different people have different experiences about screening and healthy lifestyle practices. These questions only aim to capture those. There are no correct and wrong answers. And by giving an answer you will be not subjected to disadvantage.**

***Instructions: Use predefined examples if the respondent cannot understand the given statement.***

| **30 (A). Enthusiasm on screening (For those who have a lifetime experience with screening)** | | | |
| --- | --- | --- | --- |
| State your views in relation to the following statements. | | | |
| Criteria | Not at all….10 marks | Sometimes…5 marks | Always…0 marks |
| 30.1. When you need to be fasting, have you ever forgotten to maintain fasting?  (Didn’t adhere to instructions on preparation for screening) |  |  |  |
| 30.2. For you is it difficult to think about fasting to get a screening when you do not have a disease?  (Felt demotivated to fast because asymptomatic) |  |  |  |
| 30.3. Have you postponed a planned screening test due to other priorities?  (Postponed planned screening due to other priorities) |  |  |  |
| 30.4. Have you not thought of going for another checkup though you already know about your blood sugar and blood cholesterol levels?  (Willingness for regular screening) |  |  |  |

***Instructions: Use predefined examples if the respondent cannot understand the given statement.***

| **30 (B). Enthusiasm on screening (For those who did not have a lifetime experience of screening)** | | | |
| --- | --- | --- | --- |
| State your views in relation to the following statements. | | | |
| Criteria | Not at all…..10 marks | Sometimes…5 marks | Always….0 marks |
| 30.1. Do not you like to undergo fasting for a screening test if you need to do so? |  |  |  |
| 30.2. For you is it difficult to think about fasting to get a screening when you do not have a disease? |  |  |  |
| 30.3. Have you postponed a planned screening test due to other priorities? |  |  |  |
| 30.4. Do not you like to undergo regular screening? |  |  |  |

***Instructions: Explain what is a healthy lifestyle to the respondent using the following definition.***

**A healthy lifestyle means a lifestyle that includes habits such as consuming a healthy diet (a diet with controlled salt, fat, and carbohydrates), obtaining adequate physical activities, and implementing steps to maintain recommended weight for height/waist circumference.**

| **31. Enthusiasm to initiate and maintain a healthy lifestyle** | | |
| --- | --- | --- |
| State your views in relation to the following statements. | | |
| Criteria | Yes..10 marks | No….0 marks |
| 31.1 Have you searched for information about a healthy lifestyle?  (Sought information about a healthy lifestyle) |  |  |
| 31.2. Have you ever taken the initiative to improve your lifestyle?  (Ever initiated action/s to improve healthy lifestyle) |  |  |
| 31.3 Have you ever sought advice to improve your lifestyle?  (Ever sought advice to improve a healthy lifestyle) |  |  |
| 31.4 Have you ever monitored your lifestyle?  (Ever monitored healthy lifestyle changes or improvements) |  |  |

***Instructions: Explain the following description to the respondent. Administer the given norm as it is in the questionnaire. DO NOT USE your words.***

**Note: There are many norms in the community. The following questions aim to grab your perceptions on those norms. It is just to assess the attitude pattern of the general population like you. There are no correct and wrong answers. And by giving an answer you will be not measured, stigmatised and humiliated. And these data are not shared with anyone you know or are unknown. Personal identity is not revealed.**

| **32. Acceptance on negative gender-related norms on screening, healthy lifestyle and health-seeking behaviour** | | | | | |
| --- | --- | --- | --- | --- | --- |
| State your view about following norms. | | | | | |
| Norm | Totally agree…  10 marks | Agree..10 marks | Neither agree or disagree..5 marks | Disagree..0 marks | Totally disagree…0 marks |
| 32.1. Women should look after their health as they need to manage the household and care for the children compared to men |  |  |  |  |  |
| 32.2. Women opt for government-free services as she is economical compared to men |  |  |  |  |  |
| 32.3. Women should represent all meetings at the family and community level, including health services, as she has less work compared to men |  |  |  |  |  |
| 32.4. Women should worry about diseases compared to men |  |  |  |  |  |
| 32.5. Women will accept whatever health investigation offered by the health professionals compared to men |  |  |  |  |  |
| 32.6. It will be a shame for a man to be diagnosed with a disease compared to women |  |  |  |  |  |
| 32.7. It is difficult for men to live healthy as they interact more with society compared to women |  |  |  |  |  |
| 32.8. Men try to avoid preventive health care services fearing they will be advised against habits like alcohol and tobacco use |  |  |  |  |  |
| 32.9. According to men, alcohol is the solution for every problem, including health problems |  |  |  |  |  |

***Instructions: Explain the following description to the respondent. Administer the given norm as it is in the questionnaire. DO NOT USE your words.***

**Note: There are many norms in the community. The following questions aim to grab your perceptions on those norms. It is just to assess the attitude pattern of the general population like you. There are no correct and wrong answers. And by giving an answer you will be not measured, stigmatised and humiliated. And these data are not shared with anyone you know or are unknown. Personal identity is not revealed**

| **33. Acceptance of negative norms related to NCDs and screening** | | | | | |
| --- | --- | --- | --- | --- | --- |
| State your view in relation to following norms. | | | | | |
| Norm | Totally agree…  10 marks | Agree..10 marks | Neither agree or disagree..5 marks | Disagree..0 marks | Totally disagree…0 marks |
| 33.1. NCD will come with age, so no need to bother early |  |  |  |  |  |
| 33.2. Anyone will get NCDs after 40 or 50 years. So no need to check early |  |  |  |  |  |
| 33.3. Identifying a disease leads to mental suffering |  |  |  |  |  |
| 33.4. Delaying diagnosis will reduce the time that one will have to take treatment and do lifestyle modifications |  |  |  |  |  |
| 33.5. Investigations are for the ill |  |  |  |  |  |
| 33.6. Not screening means not being diagnosed with a disease, thus healthy |  |  |  |  |  |
| 33.7. People who do early investigations are afraid of diseases |  |  |  |  |  |

***Instructions: Explain the below description to the respondent.***

**Different people have different experiences in their families about lifestyle practices. These questions only aim to capture those experiences. There are no correct and wrong answers. And by giving an answer you or your family will be not measured, stigmatised and humiliated or there will not be any disadvantage.**

| **34. Perceived family support for screening and healthy lifestyle** |  |  |  |
| --- | --- | --- | --- |
| State your view in relation to following norms. |  |  |  |
| Criteria | Yes. A lot..10 marks | Yes. A little…5 marks | No…0 marks |
| 34.1. Members of my family improved their perceived importance of NCDs (diabetes/cholesterol) |  |  |  |
| 34.2. Members of my family brought to my attention the importance of screening for diabetes/cholesterol |  |  |  |
| 34.3. Members of my family had motivated me to do a screening for diabetes/cholesterol |  |  |  |
| 34.4. Members of my family had accompanied or had made arrangements for me to undergo screening for diabetes/cholesterol |  |  |  |
| 34.5. Members of my family had encouraged or reminded me to undergo screening for diabetes/cholesterol |  |  |  |
| 34.6. Members of my family had suggested/ supported/implemented actions for a change in unhealthy dietary habits or an increase in physical activities |  |  |  |

***Instructions: Explain the below description to the respondent.***

**Different people have different perceptions of state services. These questions only aim to capture those perceptions. There are no correct and wrong answers. By giving an answer you will be not measured, stigmatised, humiliated or subjected to any disadvantage.**

***Instructions: State the following scenario to the respondent.***

**Healthy Lifestyle Centre is a freely available NCD risk factor such as high blood sugar, high blood cholesterol and high blood pressure screening service at your village PMCU/CD/BH/DH. This will function on a selected weekday (On Saturdays in some centres). You have to be fasting for 10 hours (It is advisable to be fasting after 10 pm) and you are expected to visit the centre in the morning (preferred by 8 am). The Centre will function from 8- 12 noon. You can check your blood sugar, total blood cholesterol, and blood pressure and can know your body mass index and waist circumference. For females, they can undergo a breast examination and for both males and females, there will be an oral examination. You can participate in a health education session and you will be given a date for follow-up.**

| **35. Perceived negativity on functioning** | | |
| --- | --- | --- |
| State your view in relation to the following statements. | | |
| Statement | Yes…10 marks | No…0 marks |
| 35.1. Healthy lifestyle centres, the state screening service for NCD risk factors, are for poor people |  |  |
| 35.2. It is a waste of time to utilise government health institutions for diabetes/cholesterol screening if have no severe diseases |  |  |
| 35.3. There are no experienced health professionals to detect NCD risk factors in these centres |  |  |
| 35.4. These centres are not well equipped with the necessary facilities to detect high blood cholesterol though they are located at hospitals |  |  |

| **36. Perceived quality of services in the state health sector** | |
| --- | --- |
| How can you rate the quality of services in the state health sector? | |
| Totally unsatisfied | 1 |
| Unsatisfied | 2 |
| Neutral | 3 |
| Satisfied | 4 |
| Totally satisfied | 5 |

| **37. Perceived accessibility to the HLC** | |
| --- | --- |
| How can you rate the accessibility to the HLC? | |
| Very high | 1 |
| High | 2 |
| Moderate | 3 |
| Low | 4 |
| Very low | 5 |

***Instructions: Explain the below description to the respondent.***

**Different people have different experiences in their communities regarding healthy lifestyles. These questions only aim to capture those experiences. There are no correct and wrong answers. And by giving an answer you or your community will be not measured, stigmatised and humiliated.**

| **38. Perceived community networking for NCD prevention, screening and healthy lifestyle** | | |
| --- | --- | --- |
| Have you ever heard following statements from your community? | | |
| Statement | Yes…10 marks | No…0 marks |
| 38.1. Do you know about NCD (Diabetes, high blood pressure, high blood cholesterol, heart diseases)? OR Most of the people now have NCDs. (NCDs as a common disease in the society) |  |  |
| 38.2. Do you know about the importance of screening for NCDs? OR It’s better you check early for blood sugar and cholesterol  (Importance of screening for NCD prevention) |  |  |
| 38.3. Have you done an FBS or cholesterol test? OR Do a sugar test.  (Fasting Blood Sugar or cholesterol test as key screening tests) |  |  |
| 38.4. Do you know the places available and the cost? OR You can do a test from ….for …..  (Places available to undergo screening and associated cost) |  |  |
| 38.5. Do you know the instructions to undergo a screening? OR Requirement of 8 to 10 hours fasting before withdrawing blood.  (Instructions for undergoing screening) |  |  |
| 38.6. Now your age is good to do an FBS or cholesterol test. OR Most people get diabetes at this age. (Age-related risk) |  |  |
| 38.7. Some have high values for blood sugar and cholesterol even in middle age without any symptoms. We do not know ours.  (Possibility of having a risk even asymptomatic) |  |  |
| 38.8. Reduce sugar, salt, oil intake.  (Healthy dietary practices) |  |  |
| 38.9. Do some exercise/sport/increase physical activities in your day-to-day activities (Healthy physical activities) |  |  |

| **39. Perceived presence of peer support for NCD prevention, screening and healthy lifestyle** | |
| --- | --- |
| 39.1 | Have you ever heard that your neighbours or friends in the village share their experiences/results in changing unhealthy lifestyles at least once?   1. All ways…………..1 2. Sometimes………...2 3. Rarely……………..3 4. Never………………4 |
| 39.2. | Have you ever heard that your neighbours or friends in the village appreciate others commenced healthy lifestyles at least once?   1. All ways…………..1 2. Sometimes………...2 3. Rarely……………..3 4. Never………………4 |

**Section E: Medical and screening history-related factors**

***Instructions: Ask about the situation before HLC utilisation from HLC clients***

| **40. Family history of NCDs or risk factors** | |
| --- | --- |
| Did/does at least one of your mother or father have/has the following diseases/conditions? ***(Can tick more than one answer)*** | |
| High blood sugar | 1 |
| High blood cholesterol | 2 |
| High blood pressure | 3 |
| Stroke | 4 |
| Heart attack | 5 |

| **41. Personal history of an intermediate risk factor** | |
| --- | --- |
| Do you have already been diagnosed with the following conditions? ***(Can tick more than one answer)*** | |
| High blood sugar | 1 |
| High blood cholesterol | 2 |
| High blood pressure | 3 |

***Instructions: If both 1 and 2 responses were reported, that was the end of the questionnaire. Thanks, the respondent. Otherwise, go to question 42.***

| **42. Prior experiences of screening for intermediate risk factors** | |
| --- | --- |
| Have you ever tested for blood cholesterol (fat levels in your blood)? | 1 |
| Have you ever done a test to detect fasting blood sugar? | 2 |

**End of the questionnaire. Thanks, the respondent.**
